# Evaluation of the feasibility of anomaly detection for dose management in PET examinations

**DOI:** 10.1007/s12149-025-02063-2

**Published:** 2025-06-16

**Authors:** Yusuke Fukui, Shogo Baba, Kohei Ohashi, Yukihiro Nagatani, Kazumasa Kobashi, Yoshiyuki Watanabe, Harumi Iguchi

**Affiliations:** 1 Department of Radiology, Shiga University of Medical Science Hospital, Otsu, Japan; 2 Information Technology Center, Seinan Gakuin University, Fukuoka, Japan

**Keywords:** PET-CT, Anomaly detection, Dosimetry, Unsupervised learning, Radiation dose management

## Abstract

**Objective:**

Owing to the revision of the Medical Care Act in 2020, managing and recording radiation doses in PET-CT examinations have become mandatory. In this study, we investigated unsupervised anomaly detection methods as a potential solution to minimize input errors in dose recordings.

**Methods:**

We analyzed data extracted from our database, including patient body weight, positron emission tomography (PET) dose, and dose length product (DLP). Several anomaly detection models, such as one-class support vector machine (OCSVM), Hotelling's T2 method, multivariate statistical process control (MSPC), isolation forest, and local outlier factor (LOF), were applied and compared. The dataset included 3509 entries for model training and 499 entries for evaluation. Anomalies that could be potential input errors were evaluated using metrics, such as precision, recall, *F*1 score, receiver operating characteristics-area under the curve (ROC-AUC), and precision–recall-AUC (PR-AUC).

**Results:**

We demonstrated that Hotelling's T2 method and MSPC's *T*^2^ statistic outperformed other models, achieving a recall of 1.0 and AUCs of 1.0, effectively detecting input errors in radiation dose records. Furthermore, our findings suggest that unsupervised anomaly detection can not only identify input errors but also detect excessively high or low radiation doses, contributing to improved dose management in PET-CT examinations.

**Conclusion:**

These findings suggest that unsupervised anomaly detection is a promising approach to improve the accuracy of dose management in PET-CT examinations, enhancing patient safety and compliance with regulatory standards.

## Introduction

Following revisions to Japan's Medical Care Act Enforcement Regulations on April 1, 2020, managing and recording exposure doses have become mandatory for medical devices used in diagnostic radiology, including computed tomography (CT) X-ray equipment and positron emission tomography (PET) with radioactive isotopes. Diagnostic reference levels (DRLs) are recommended for the evaluation and the optimization of exposure doses [1].

In dose management for PET examinations using positron-emitting radioactive isotopes, it is common to combine CT scans for attenuation corrections. In this series of examinations, the administered dose of PET pharmaceuticals and the exposure from CT scans were the subjects for management.

In Japan, the DRL was originally published by the Japan Network for Research and Information on Medical Exposure (J-RIME) in 2015 and updated in 2020 [2–4]. Between these updates, attempts were made to investigate the appropriate doses in specific regions [5]. Dose optimization efforts, such as evaluating image quality by categorizing CT purposes and body weight in PET-CT examinations, are ongoing [6–9]. With advancements in imaging equipment and reconstruction methods, the exposure doses can be further reduced. We are also required to re-optimize doses when introducing new equipment or when certain events occur at each facility. Such cases and regular reviews are needed in future, and continuous dose management and evaluation are essential.

Currently, in Japan, exposure doses from CT scans are output in the Radiation Dose Structured Report (RDSR) format as predefined indices like the Computed Tomography Dose Index (CTDI) and the Dose Length Product (DLP) from many imaging devices. Recently, RDSR outputs from PET pharmaceutical administration devices have become available. However, not all facilities have complete data integration among PET-CT imaging devices, PET pharmaceutical administration devices, and radiology information systems (RIS) [2]. In such facilities, manual data entry by staff is necessary when centrally managing PET-CT exposure doses. Manual input in dose management can lead to errors, and recording incorrect data is inappropriate, making dose optimization based on such data difficult.

The exposure dose from CT scans using auto exposure control (AEC) correlates with body weight [10]. It is common to determine the administered dose of PET pharmaceuticals based on body weight or lean body mass [4, 7]. Therefore, using machine learning and anomaly detection methods based on data from three variables—the administered dose of PET pharmaceuticals (MBq), DLP (mGy cm), and body weight (kg)—we considered that it could effectively detect input errors.

This study aims to investigate the feasibility of unsupervised anomaly detection methods based on PET dose, DLP, and body weight, with the goal of minimizing input errors in dose recordings and thereby contributing to the accurate determination of appropriate exposure doses for examinations.

## Materials and methods

### Dataset

Since our institution cannot directly output information from the administration device, we centrally manage data such as exposure doses using FileMaker™ (Claris International Inc.). In FileMaker, patients input their body weight by themselves in advance, and radiological technologists responsible for the examination input the actual administered PET dose and DLP after the examination. In exposure dose management, manual input errors are minimal compared with the total number of examinations, and it is expected that the majority of data are correctly entered. However, there is a possibility of input errors for all variables. In this study, we targeted the examination data from January 6, 2020 to October 31, 2023, during which dose management was conducted using FileMaker.

The learning model was constructed using unsupervised learning for reasons described later. However, labels are necessary when evaluating the model. Therefore, we divided the data into 3509 cases from January 6, 2020 to April 27, 2023 as training data and 499 cases from the subsequent half-year until October 31, 2023 as test data. In the test data, we checked the input data in FileMaker against the data recorded in the RIS, which was confirmed by a dedicated pharmacologist on the day following each examination, and assigned labels indicating input errors (anomaly labels) to the data considered to have input errors. Anomaly labels were assigned by two professionals in consensus: a nuclear medicine specialist technologist and a medical physicist engaged in nuclear medicine.

In this study, computations were performed on a computer equipped with Windows 11 Home 64-bit OS (Microsoft Japan; Tokyo), an Intel(R) Core(TM) i5-10210U CPU @ 1.60 GHz 2.11 GHz (Intel Corporation, Santa Clara, CA, USA), and 8.00 GB of RAM. The implementation environment used a Jupyter Notebook, and the frameworks included Python libraries PyOD (1.0.9) and scikit-learn (1.3.0), executed in an Anaconda environment. Additionally, all examinations included in the training and test data were performed using Discovery PET-CT D710 (GE Healthcare Japan; Tokyo, Japan). The imaging conditions included a tube voltage of 120 kV, and the dose used AEC with a default noise index setting of 35. Either FDGscan® Injection (Nihon Medi-Physics Co. Ltd, Tokyo, Japan) or Fludeoxyglucose (18F) (PDRadiopharma Inc., Tokyo, Japan) was used as the FDG-PET radiopharmaceutical. The dose was selected based on the patient’s body weight from five fixed levels: 111, 148, 185, 222, or 259 MBq at the calibration time. The actual administered dose was estimated by adjusting for the time difference between the calibration time and the actual administration time.

### Method selection

Labeling all the data used for training to build an anomaly detection model is cumbersome and costly. Therefore, it is practical to use unsupervised anomaly detection methods that do not require labeling. Various unsupervised learning methods have been advocated for anomaly detection purposes [11–13]. Since the data in this study are not complex high-dimensional data like images or text, we decided to use simple models rather than complex methods utilizing deep learning.

Anomaly detection methods can be broadly divided into clustering-based methods, methods that consider data deviating from a normal distribution as anomalies using probability distributions, methods that judge anomalies based on reconstruction errors, and methods that assess anomalies based on the distances between individual data points [13]. While some methods may be difficult to use when the amount of data or number of features is large, the data considered in this study have only three features, making the computation feasible. While simple statistical approaches such as regression analysis can be used to detect anomalies when there is a clear dependent relationship between variables, these methods are limited in flexibility. In our dataset, which includes PET dose, DLP, and body weight, no single variable serves as an explicit dependent variable, and the relationships among variables may be nonlinear. Therefore, a single regression model is unlikely to effectively capture potential anomalies. Therefore, we adopted five methods, consisting of:

1. One-Class Support Vector Machine (OCSVM) (clustering-based method).
2. Hotelling’s T2 method (probability distribution-based method).
3. Multivariate Statistical Process Control (MSPC) (reconstruction error-based method).
4. Isolation Forest (distance-based method).
5. Local Outlier Factor (LOF) (distance-based method).

We then measured anomaly scores for each data point and compared the detectability of anomalous data. Notably, the MSPC method yields two separate anomaly scores—the *T*^2^ statistic and the *Q* statistic—resulting in a total of six anomaly indicators evaluated across the five methods. We believe that comparing multiple anomaly detection methods can provide valuable insights for developing a more comprehensive and robust approach to identifying a wide range of anomalies.

#### One-class support vector machine (OCSVM)

One clustering-based method is the OCSVM [14], which is based on the Support Vector Machine (SVM), a classification method that classifies data into two or more classes.

In the OCSVM, a single class is created with a spherical boundary, and data points outside this sphere are judged as anomalies. An optimization problem is solved to make the sphere enclosing the training data as small as possible. However, because the data include noise, it is difficult to create a sphere that contained all the data points. Therefore, instead of strictly minimizing the radius, an optimization problem that allows for some margins is solved. The equations are as follows: where $r$ is the radius of the sphere, $b$ is the center of the sphere, $u_{n}$ is the slack variable representing the allowable margin, and the optimization problem is solved for each individual data point $n$ out of $N$ data points. $C$ is a constant called the regularization constant that determines tolerance.

$${\text{minimize}}_{{r^{2} ,b}} \left( {r^{2} + C\mathop \sum \limits_{n = 1}^{N} u_{n} } \right)$$

$${\text{subject}}\;{\text{to}}\quad \parallel x_{n} - b\parallel ^{2} \le { }r^{2} + { }u_{n}$$

"Subject to"represents the constraint condition, and under this condition, the optimization problem is solved to find the center b and radius $r$. The anomaly score $\alpha \left( x \right)$ of the data is defined as the distance from the decision boundary and can be expressed as follows: the distance from the decision boundary exists even inside the sphere, but positive values, that is, distances outside the boundary, indicate the anomaly score.

$$a\left( x \right) = \parallel{ }x - b\parallel^{2} - { }r^{2}$$

Kernel functions can be used for inner-product calculations to consider the nonlinearity between variables in SVM calculations. In this study, we used the default Radial Basis Function (RBF) kernel [15, 16]. The hyper-parameter $\gamma$, which controls the complexity of the RBF kernel, and $\nu$, which regulates the trade-off between the model's sensitivity to outliers and the flexibility of the decision boundary were optimized via grid search using the area under the receiver operating characteristic curve (ROC-AUC) as the evaluation metric. As a result, $\gamma$ and $\nu$ were set to 1e−5 and 0.01, respectively.

Although the parameter $C$ is often used in standard SVM classification to control the margin and misclassification penalty, OCSVM uses $\nu$ instead. Therefore, in the implementation, $\nu$ was used in place of $C$.

#### Hotelling’s T2 method

We used Hotelling’s T2 method as a probability distribution approach, applicable when the training data are assumed to be independent samples following a single multivariate normal distribution [16, 17]. The parameters of this distribution, namely the mean vector and the covariance matrix, estimated using the maximum likelihood method based on the training data, and were assumed to represent the distribution of normal data. New data were regarded as anomalies if they significantly deviated from the estimated mean.

Since the variance differs across variables, Hotelling’s T2 method accounts for these differences. Furthermore, to consider the correlations between variables, we used the inverse of the variance–covariance matrix and defined the anomaly score $T^{2}$ as follows:

$$T^{2} = \left( {x - \mu } \right)^{T} { }\Sigma^{ - 1} { }\left( {x - \mu } \right)$$

When each variable followed a multivariate normal distribution, the anomaly score of the data followed a chi-square distribution [16]. Therefore, the data with high $T^{2} { }$ values were considered abnormal.

#### Multivariate statistical process control (MSPC)

Principal component analysis (PCA) is a fundamental linear dimensionality reduction method. While there are anomaly detection methods utilizing PCA, we employed MSPC in this study, which applies PCA and is widely used in areas like production management [18]. The PCA seeks a subspace by maximizing the variance of the training data. The transformation of data into a subspace is performed through linear combinations that consider the correlations between the original variables [19]. In the MSPC, by applying Hotelling’s T2 method to the values in this subspace, the distance from the mean under consideration of correlations can be obtained as the $T^{2}$ statistics. Furthermore, the squared difference between the values transformed by PCA ${ }(\hat{x}_{m} )$ and the original values $(x_{m} )$ is called the reconstruction error and is defined as follows:

$$Q = { }\mathop \sum \limits_{m = 1}^{M} \left( {x_{m} - { }\hat{x}_{m} } \right)^{2}$$

This reconstruction error is known as the $Q$ statistic. Because PCA utilizes correlations between variables, data with large reconstruction errors are considered abnormal data that deviate from these correlations. In MSPC, $T^{2}$ and $Q$ statistics were obtained as measures of anomalies [18]. Accordingly, each variable was analyzed separately to detect anomalies on an individual basis. This study performed the PCA transformation from a 3-dimensional space to a 2-dimensional subspace as there were three original variables.

#### Isolation forest

The first distance-based method is an isolation forest that uses decision trees [20]. In this method, it is assumed that anomalous data have properties different from those of normal data and that there are very few similar data. In decision trees created under such assumptions, anomalous data can be easily isolated without requiring many splits based on various conditions. In other words, if the path length from the root of a decision tree to a terminal node is short, the anomaly score is considered to be high. Isolation forest builds multiple such decision trees, similar to random forests, and the final anomaly score is calculated as the average of the scores from all trees.

For the isolation forest, all parameters were set to their default values. For example, the number of trees (n_estimators) was 100, the number of samples used to train each tree (max_samples) was set to"auto", the expected proportion of anomalies (contamination) was set to 0.1, and the number of features used to split each node (max_features) was set to 1.0.

#### Local outlier factor (LOF)

Another distance-based method is the LOF [21], which considers the density of data.

This method assesses whether the target data are located in a region with a lower density than the surrounding data. Data in such low-density regions are considered to have properties different from those of the surrounding data and are thus judged to be anomalies. Various distance metrics can be used to measure the distance between target data and surrounding data. Among them, this study used the most common metric, the Euclidean distance, with the number of neighbors (n_neighbors) set to 50.

### Determination of thresholds

In all methods, the anomaly score is obtained as a statistical measure of a continuous variable. Therefore, a threshold is necessary to determine whether the data were anomalous or normal. We adopted the quantile method, which determines the threshold based on the proportion of all anomalous data, and the labeling method, which directly sets the anomaly score of the anomalous data as the threshold [22]. We defined the smaller value of the two candidates as the final threshold.

**Fig. 1:**
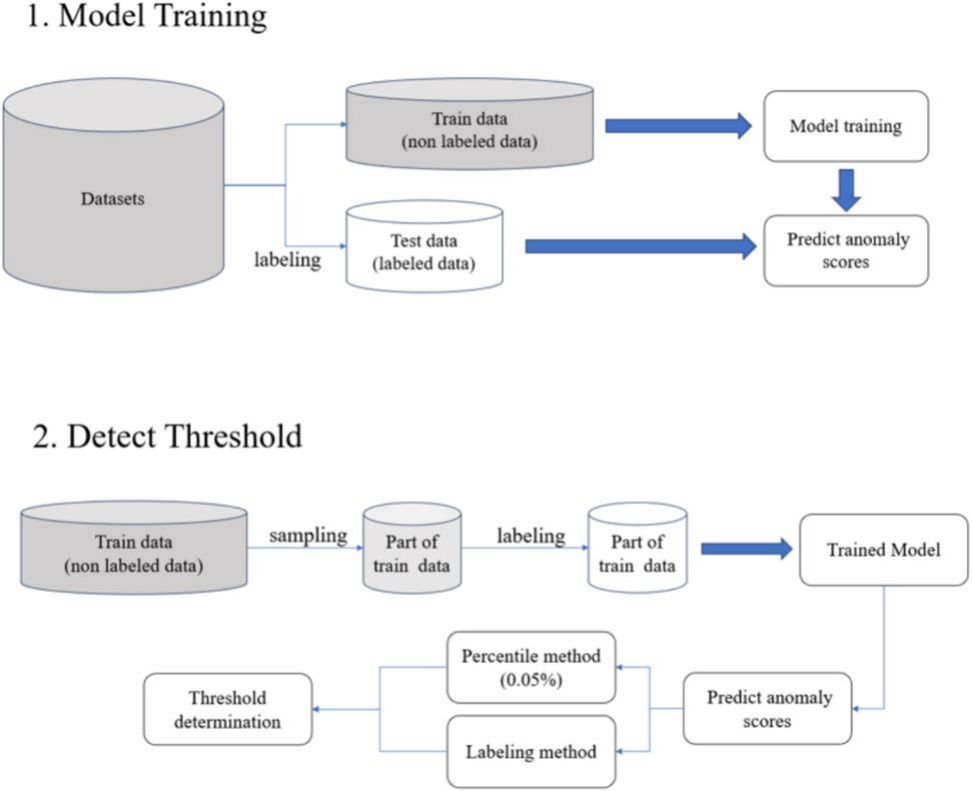
Process of model training and anomaly threshold-setting

The threshold used for evaluating the test data was determined using 685 data points from January 6, 2020 to September 30, 2020, which were a portion of the training data labeled for input errors in the same procedure as the test data. Concretely speaking, for the labeling of anomalous data points, the accuracy of the data in RIS was first confirmed by a pharmacist on the day following each examination. Based on this verified information, all three variables recorded in FileMaker—PET dose, DLP, and body weight—were reviewed independently to identify input errors, in consensus by a nuclear medicine specialist technologist and a medical physicist. Only data points with confirmed input errors were labeled as anomalies. There were two anomalous data within the data for threshold determination, and Table 1 details the anomalous data used for the evaluation and threshold determination. In the quantile method, we set the quantile to 0.3%, the proportion of anomalous data in the threshold determination data. In the labeling method, the lowest anomaly score among the anomalous data was set as the threshold for each method. Figure 1 illustrates the overall flow of the model training and threshold determination.

**Table 1:** Details of anomalies in each dataset

Data point	638 [threshold_dataset]	678 [threshold_dataset]	155 [test_dataset]	421 [test_dataset]
PET dose(MBq)	26.5	150.6	**2040.0**	306.0
DLP(mGy cm)	**174.50**	98.28	209.30	**27,799.00**
Weight(kg)	4.920	**448.000**	58.100	84.201

Bold text indicates outliers

### Model evaluation metrics

In anomaly detection, the most common evaluation metric is the receiver operating characteristic (ROC) curve evaluation [22]. The area under the curve (AUC) serves as an evaluation of a model that does not depend on a threshold. Because the data were imbalanced, we also evaluated the area under the precision–recall curve (PR-AUC) to account for this effect. Additionally, since the thresholds are determined for each model, an evaluation using a confusion matrix is also possible. In this study, we prioritized recall, but if there are too many false positives, anomaly detection becomes meaningless; therefore, we also evaluated precision and the F1 score in addition to recall. Here, true positive (TP) is the number of correctly identified anomaly labels, false positive (FP) is the number of normal labels incorrectly judged as anomalies, and false negative (FN) is the number of anomaly labels incorrectly judged as normal.

$${\text{Precision}} = \frac{{{\text{TP}}}}{{{\text{TP}} + {\text{FP}}}}$$

$${\text{Recall}} = \frac{{{\text{TP}}}}{{{\text{TP}} + {\text{FN}}}}$$

$$F1\;{\text{score}} = \frac{{2{\text{TP}}}}{{2{\text{TP}} + {\text{FN}} + {\text{FP}}}}$$

## Results

**Fig. 2:**
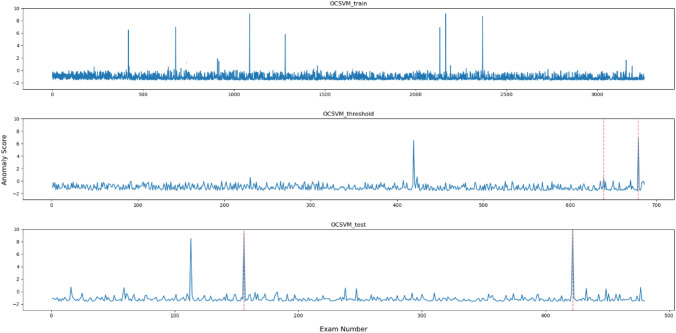
Outliers identified for each data point using OCSVM

**Fig. 3:**
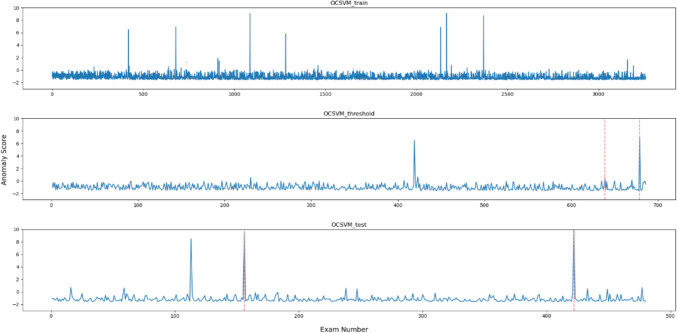
Outliers identified for each data point using Hotelling's T2 method

**Fig. 4:**
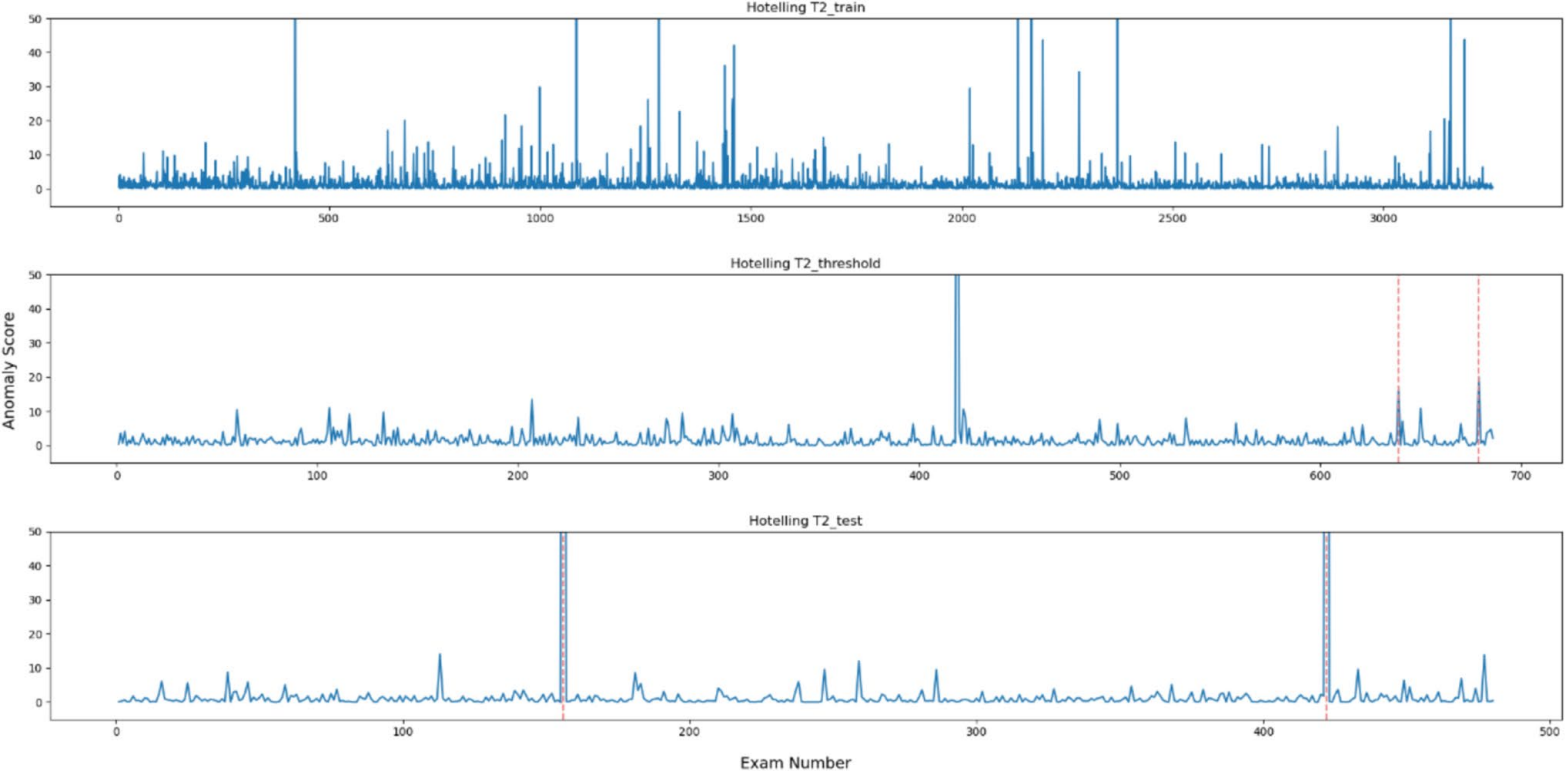
Anomalies detected by ${ }Q$ statistics for each data point using the MSPC method

**Fig. 5:**
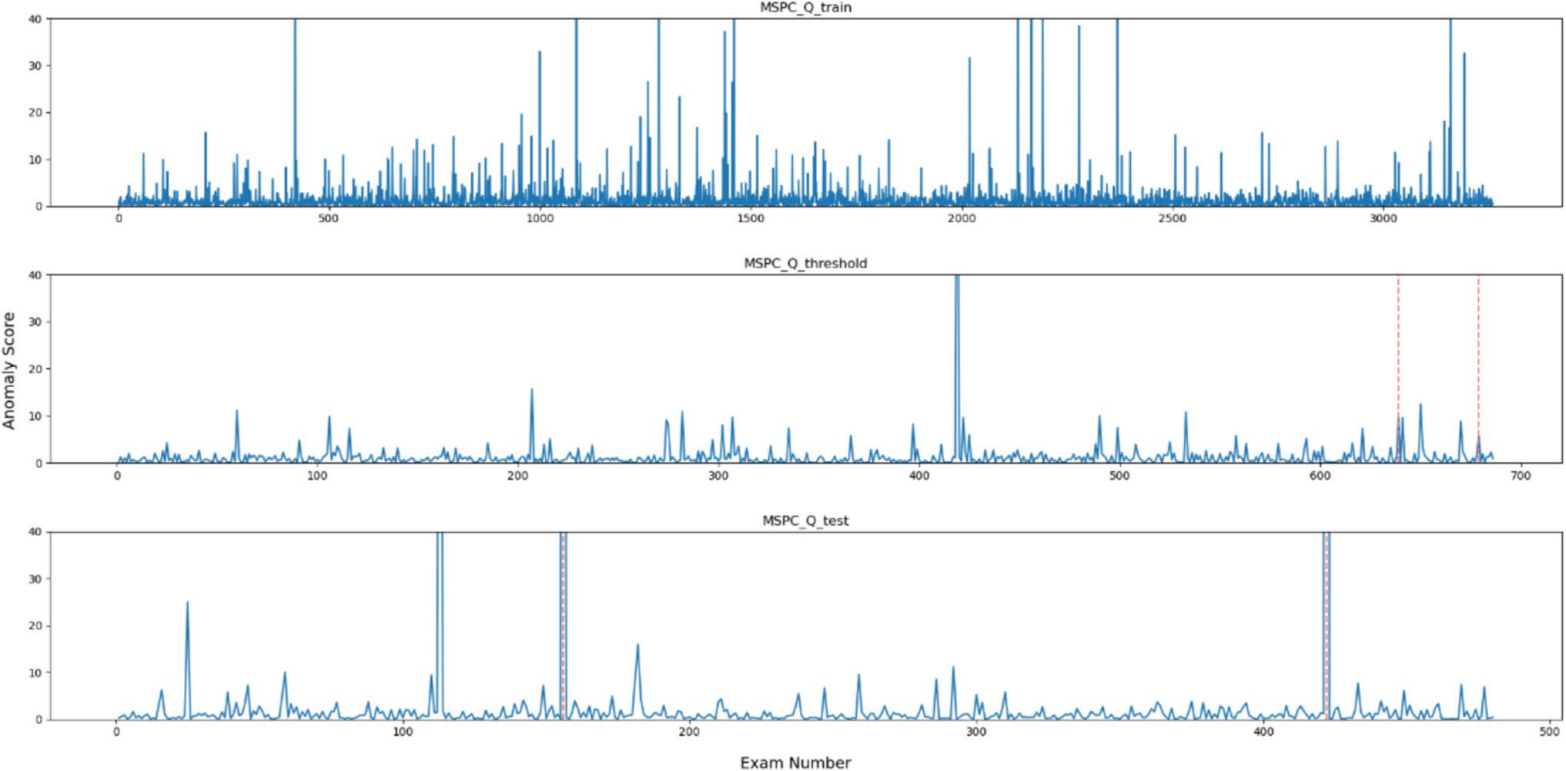
Anomalies detected by $T^2$ statistics for each data point using the MSPC method

**Fig. 6:**
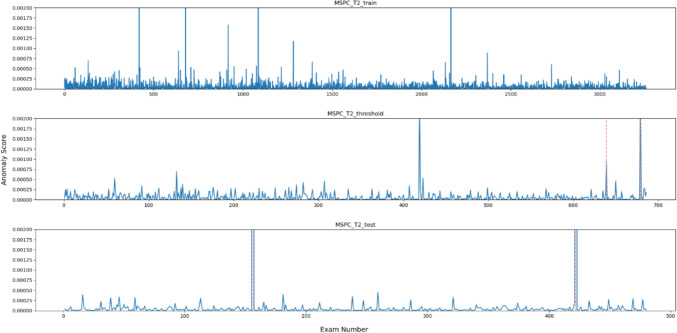
Anomaly score for each data point using the isolation forest method

**Fig. 7:**
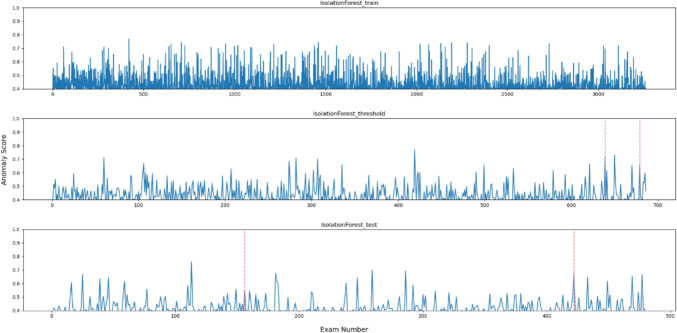
Degree of anomaly for each data point using the LOF method

Figures 2, 3, 4, 5, 6 and 7, respectively, show the statistical measures representing the anomaly scores of the learning data, test data, and data used to determine the thresholds. In each dataset, *x*- and *y*-axes of the figures represent the examination number assigned to individual examinations and the anomaly score of each model, respectively. In the threshold determination data, No. 638 and 678, and in the test data, No. 155 and 421 are identified as anomalous data, indicated by dotted red lines in the graphs. Table 2 lists the thresholds and anomaly scores for the anomalous data in each model.

**Table 2:** Thresholds for each method and anomaly scores for anomalous data

Method name (threshold)	638 [threshold_dataset]	678 [threshold_dataset]	155 [test_dataset]	421 [test_dataset]
OCSVM (0.4524)	0.4524	6.9392	9.6560	10.1218
Hotelling T2 (17.2034)	17.2034	20.1000	447.1381	478.8364
MSPC-T2 (0.000659)	0.000938	0.002835	0.094820	0.602327
MSPC-Q (5.8456)	10.0969	5.8456	521.4459	96,073.3197
IsolationForest (0.6590)	0.7291	0.6590	0.5574	0.7014
LOF (3.2775)	3.4378	22.6516	68.7054	207.6386

In the five methods excluding the isolation forest, the anomaly scores of the anomalous data in the test data were higher than the other data. However, in the data for threshold determination, the anomaly scores were not always higher in the isolation forest and MSPC's $Q{ }$ statistics and in other methods. In contrast, the anomaly score of the second anomalous dataset, No. 678, was high, and the anomaly score of the first anomaly, No. 638, was low. Moreover, in the isolation forest, there were some normal data with high anomaly scores in both learning and test datasets. In the other methods, there were some data with higher anomaly scores than those with anomaly labels.

The results of precision, recall, F1 score, PR-AUC, and ROC-AUC for each method are shown in Table 3. The AUC values of the test data were equivalent across the methods except for the isolation forest. In this study, the most important metric was recall. Except for the isolation forest, the recall was 1.0, detecting both anomalous data. MSPC's $T^{2}$ statistics and Hotelling’s T2 showed favorable results with all three metrics being 1.0.

**Table 3:** Results for each evaluation index by method

	Precision	Recall	F1 score	PR-AUC	ROC-AUC
OCSVM	0.222	1.000	0.364	1.000	1.000
Hoteling T2	1.000	1.000	1.000	1.000	1.000
MSPC-T2	1.000	1.000	1.000	1.000	1.000
MSPC-Q	0.105	1.000	0.190	1.000	1.000
Isolation Forest	0.111	0.500	0.182	0.151	0.971
LOF	0.667	1.000	0.800	1.000	1.000

## Discussion

In our institution, in accordance with the guidelines of nuclear medicine [4, 23], the administered dose is calculated based on body weight. Moreover, a strong positive correlation between body weight and DLP in the training data (*r* = 0.7456) and the test data (*r* = 0.7456) was demonstrated in this study. Therefore, we hypothesized that the MSPC method, which considers correlations, and Hotelling’s T2 method, which accounts for the variance and covariance among individual variables, would be effective anomaly detection methods. As expected, the results confirmed that these methods are suitable for this task. On the other hand, the precision of the $Q$ statistics in the MSPC was lower compared to the $T^{2}$ statistics of the MSPC. When data were compressed from three to two dimensions using PCA, the cumulative contribution rate, representing the amount of information retained in the 2-dimensional data, was 82.1%. A cumulative contribution rate of over 80% indicates that most of the information was preserved during the data transformation. These results imply that methods relying solely on reconstruction error are not sufficient for this task. Moreover, because the threshold was set to match the lowest anomaly scores assumed for anomalous data, the increase in false positives led to a decrease in precision and the F1 score.

All the metrics for isolation forest were generally low, and anomaly scores of the anomalous data were similar to those of the non-anomalous data as shown in Fig. 6. Because administered PET dose can vary among cases even with the same body weight depending on the scheduled examination time and DLP changes according to differences in data acquisition range, classification method based on conditional branching, such as decision tree, was shown to be unsuitable for this task. The OCSVM theoretically corresponds to more complex classification compared with the other methods, as it creates nonlinear boundaries. However, in this method, the anomaly scores of the data for threshold determination were low possibly due to overfitting or underfitting, leading to a lower threshold and a decreased precision, indicating that OCSVM is not appropriate as the anomaly detection method for task like this. While there is potential for performance improvement by adjusting the kernel function and hyper-parameters, it is more efficient for this task to select other simple methods that consider correlations. Judging from the anomaly scores, in each method like Hotelling’s T2, MSPC's $T^{2}$, and LOF, can easily separate the anomalous data and can be considered as feasible methods. Especially, either Hotelling’s T2 or MSPC's $T^{2}$, with values of 1.0 for the three metrics; precision, recall and *F*1 score, is desirable.

In a non-anomalous case, No. 112 in the test data, the anomaly score was considerably high in the five methods except for MSPC's $T^{2}$statistic. Patient's body weight and DLP were 107.5 kg and 1467 mGy cm, respectively. At our institution, when the noise index is set to 35, the standard deviation (SD) of the liver was about 30 and the DLP was about 540 mGy cm for cases of similar weight and habitus. This dose level, with a noise index of 35, can roughly correspond to about half of the DRL value as FDG examinations are generally diagnosed based on conventional CT series for configurational and anatomical information. However, because no prior CT examination was performed in this case, the noise index was actually set low, and the SD of the liver was about 15. In another non-anomalous infant case, No. 418 in the data for the threshold determination, the anomaly score was considerably high in the five methods except for isolation forest. Infant's body weight and DLP were 16.3 kg and 1086.3 mGy cm. In this case, the use of CT imaging data was necessary for radiotherapy planning, which led to the application of a fixed dose and resulted in an overdose. Thus, these anomaly detection methods can find cases with excessive dose and may provide a clue to discuss CT dose optimization in unusual situations with alternative CT data acquisition for radiation therapy planning and diagnostic radiology. Comparing the two methods calculating the $T^{2}$ statistics in this regard, only Hotelling’s T2 method demonstrated a higher anomaly score in both cases, and may be a more useful method for detecting input error as well as excessive dose. In addition, these anomaly detection methods could play a crucial role in the optimization of DRL diagnostic level through their ability to exclude cases with extraordinary doses among mass data collected from nationwide institutions.

An analysis of anomaly scores revealed that many input errors differed from intended values by several orders of magnitude. Therefore, a simple approach using univariate statistical process control (USPC), which sets upper and lower limits for each variable, could potentially detect a large number of input errors. For instance, USPC successfully identified specific input errors, such as Case No. 678 in the data for the threshold determination and Cases No. 155 and No. 421 in the test dataset. However, it does not guarantee the identification of all anomalies. As shown in Table 1, Case No. 638 in the data for the threshold determination was not detected by USPC because its DLP value was nearly equivalent to the normal range for adults. This limitation arises from USPC's use of a single threshold value for each variable, including PET dose, DLP, and body weight. In particular, when thresholds are determined based on mean values of the general population, the measurements for pediatric patients and individuals with larger body habitus considerably deviate from those for adults with an average body habitus, making accurate error detection challenging. An alternative approach involves dividing each variable into specific ranges and determining the normal range for other variables within each range. For example, Table 4 demonstrates the mean values of PET dose and DLP calculated in the training datasets divided by 10 kg weight intervals. By setting normal ranges for these finer subdivisions, more precise anomaly detection can be achieved. However, in cases where data for patients outside the normal range of body habitus are limited (e.g., under 10 kg and over 80 kg ranges in Table 4), a small number of errors can significantly affect the mean values, making stable anomaly detection difficult. Therefore, it is crucial to consider the interrelationships among PET dose, DLP, and body weight as three-dimensional data. Our proposed method incorporates these relationships to achieve more effective error detection. Furthermore, beyond detecting input errors, it is also essential to account for variable interdependencies to identify cases of excessive or insufficient dose administration.

**Table 4:** Mean values of PET dose and DLP by 10 kg weight intervals in the training datasets

Weight range (kg)	Average PET dose (MBq)	Average DLP (mGy cm)
[0, 10)	49.21	43.88
[10, 20)	71.14	105.25
[20, 30)	103.24	63.97
[30, 40)	175.64	92.26
[40, 50)	204.76	128.84
[50, 60)	233.23	173.23
[60, 70)	257.99	222.59
[70, 80)	288.68	269.49
[80, 90)	307.88	534.75
[90, 100)	329.58	365.99
[100, 110)	338.93	542.17
[110, 120)	316.50	634.00
[120, 130)	343.00	754.04

Table 5 lists the cases identified as anomalies by Hotelling’s T2 method or MSPC’s *T*^2^ within the training dataset, along with several different reasons. Specifically, these cases included delays in FDG administration (due to preprocessing delays), required rescans of CT images (caused by patient movement), and significant increases in CT dose (due to changes in noise setting for the AEC for overweight patients). Although CT dose is controlled by the AEC, many cases with excessive radiation exposure demonstrated in Table 5 involved DLP values that were approximately twice as high as those of the examinations that the identical patients underwent on a different day. These findings intuitively reflect intended dose overadjustments rather than adjustments based on the noise index for overweight patients. The dose did not deviate from the standard value within the same order of magnitude, but it was significantly higher compared to other patients with similar body habitus. Furthermore, USPC, a simple filtering method, cannot adequately account for the interdependencies among multiple variables, which makes it difficult to establish appropriate normal ranges, even in three-dimensional data. In contrast, anomaly detection methods such as Hotelling’s T2 method enable an integrated evaluation of these variables, allowing for more consistent and reliable anomaly detection.

**Table 5:** Cases identified as anomalies by Hotelling’s T2 or MSPC’s *T*^2^ in the training dataset and their respective reasons

PET dose (MBq)	DLP (mGy cm)	Weight (kg)	Hoteling T2	MSPC-T2	Reason for detection
98.3	239.11	64.9	0.000696	9.8438	FDG administration with much lower radiation due to order of delivered radioisotope agents with inadequate radio-activity
77.8	1086.28	16.3	0.002394	176.6644	Application of a fixed higher dose without AEC
26.5	174.50	4.92	0.000938	17.2035	Mistakenly input of DLP value for another patient
150.6	98.28	448.0	0.002835	20.1000	Mistakenly input of body weight
34.2	273.34	77.9	0.001576	21.6915	Unachieved FDG administration at the scheduled time due to the failure to comply dietary restrictions
317.5	568.00	72.0	0.000001	18.4427	Rescanned CT image due to the failure to sustain body posture
350.6	672.47	91.3	0.000003	29.8501	Dose elevation up to approximately twice of the dose at standard examination by dose overadjustment than the adequate noise index
1765	233.23	44.2	0.068416	889.3061	Mistakenly input of dose
309.8	565.28	84.1	0.000002	18.3588	Rescanned CT image due to the failure to sustain body posture
316.8	634.74	88.5	0.000007	26.1904	Rescanned CT image due to the failure to sustain body posture
180.2	1103.00	40.0	0.001178	151.2338	CT examination at higher doses to be used for treatment planning
314.7	605.81	81.6	0.000002	22.7373	Dose elevation up to approximately twice of the dose at standard examination by dose overadjustment than the adequate noise index
86.2	232.21	40.6	0.000663	11.0133	Unachieved FDG administration at the scheduled time due to the failure to comply dietary restrictions
333.9	712.89	105.0	0.000013	36.1732	Dose elevation up to approximately twice of the dose at standard examination by dose overadjustment than the adequate noise index
347.9	618.49	86.9	0.000011	23.2538	Dose elevation up to approximately twice of the dose at standard examination by dose overadjustment than the adequate noise index
253.7	604.52	75.0	0.000125	26.4661	Considerable dose elevation by dose overadjustment than the adequate noise index
343	754.04	123.4	0.000027	42.1039	Considerable dose elevation by dose overadjustment than the adequate noise index
337.4	667.02	112.5	0.000006	29.5503	Considerable dose elevation by dose overadjustment than the adequate noise index
292.8	1222.45	109.0	0.000655	162.9579	Considerable dose elevation by dose overadjustment than the adequate noise index
258.1	169.77	5556.3	0.322500	3216.3752	Mistakenly input of the patient weight
342.1	763.39	111.7	0.000019	43.6497	Dose elevation to obtain alternative CT images due to noise level reduction because of missed conventional CT images prior to FDG-PET examination
363.3	707.75	100.3	0.000004	34.3020	Dose elevation to obtain alternative CT images due to noise level reduction because of missed conventional CT images prior to FDG-PET examination
306.8	1585.71	72.1	0.000881	300.6631	CT examination at higher doses to be used for treatment planning
243.7	529.51	66.4	0.000098	18.2217	Rescanned CT image due to the failure to sustain body posture
274	569.36	69.5	0.000038	20.5959	Rescanned CT image due to the failure to sustain body posture
263.7	556.69	65.8	0.000051	19.8249	Considerable dose elevation by dose overadjustment than the adequate noise noise index
307.3	837.40	89.2	0.000102	59.7736	Rescanned CT image due to the failure to sustain body posture
236.7	706.45	57.2	0.000230	43.7964	Rescanned CT image due to the failure to sustain body posture

In this study, we demonstrated that anomaly detection methods based on multivariate relationships among the three variables—body weight, DLP, and administered PET dose—can effectively detect input errors and excessive doses that are often overlooked by simple threshold-based approaches. In particular, Hotelling’s T2 method and *T*^2^ statistics of MSPC showed high detection accuracy by incorporating multivariate analysis that considers correlations, and their applicability in clinical practice is expected.

However, this study has several limitations. The dataset was limited to PET examinations conducted at a single institution, and the number of anomaly cases (i.e., input errors) in the test data was only two. Although we split the data chronologically to ensure external validity, the model has not yet been evaluated using data from other institutions. These points suggest limitations in assessing generalizability and robustness of the proposed method.

In future work, it will be necessary to construct a more generalizable and clinically applicable anomaly detection model by evaluating the method using datasets collected from multiple institutions and including various types of input errors and excessive dose cases.

In addition, while the Hotelling’s T2 method demonstrated both strong performance and ease of implementation, it also tends to yield higher anomaly scores for patients whose body habitus deviate significantly from the average due to its dependence on deviations from the mean value. Therefore, in practical applications, it is important to incorporate procedures, such as manual review of detected anomalies and adjustment of decision thresholds tailored to the patient population, at each facility.

## Conclusion

Monitoring dose data using unsupervised anomaly detection methods can detect information input errors regardless of body habitus. Furthermore, these feasible methods could play a crucial role in optimizing DRL through the ability to exclude these cases with extraordinary doses and other anomalies.

## Data Availability

The data that support the findings of this study are not openly available due to reasons of sensitivity and are available from the corresponding author upon reasonable request. Data are located in controlled access data storage at Shiga University of Medical Science Hospital.
